# Data on the estimation of thermomechanical damage for fired clay bricks

**DOI:** 10.1016/j.dib.2021.106997

**Published:** 2021-03-26

**Authors:** Léon Arnaud Mpoung, Jean Calvin Bidoung, Jordan Valdès Sontia Metekong, Basil Yilareng Suila, Jean Raymond Lucien Meva'a

**Affiliations:** a Laboratory of Civil Engineering and Mechanics, National Advanced School of Engineering of Yaoundé, University of Yaoundé 1, P.O. BOX 8390, Yaoundé, Cameroon; b Local Materials Promotion Authority, P.O. Box 2396, Yaoundé, Cameroon; c National Committee for Technology Development (CNDT), P.O. BOX 1457, Yaoundé, Cameroon

**Keywords:** Clay, Thermomechanical (TM) damage, Unified strength theory (UST) criterion, Fired clay bricks

## Abstract

Fired bricks are on high demand in building constructions because of their cheapness, appearance, robustness, isolation achievement and sustainability. To make fired bricks, Constructions and eco-friendly sector used clay materials. However, the major challenge in their utilization is their thermal and mechanical behavior after exposure. Problems occur mainly when permanently subjected to increased temperature which severely influence its durability, and in this case an overall failure mode calculation is essential. In this work a simple approach based on the Unified Strength Theory (UST) criterion was used to estimate the thermomechanical damage. Results of thermomechanical damage values are shown.

## Specifications Table

**Table:** 

Subject	Engineering
Specific subject area	Materials, Damage mechanics
Type of data	Tables, Figures and Process description
How data were acquired	The X-ray diffraction (XRD) were recorded in the range 2 ° ≤ 2θ ≤ 70 ° with a diffractometer Bruker Advance Eco equipped with a Cu Kα1 (λ = 1.5418 Å) radiation source. The thermogravimetric and thermal differential analysis (TG/DTA) was performed on the Automatic Multiple Sample Thermogravimetric Analyzer TGA-2000. The chemical analysis of major elements (SiO_2_, Al_2_O_3_, TiO_2_, Fe_2_O_3_ total, MnO, MgO, CaO, Na_2_O, K_2_O, P_2_O_5_, LOI) were assayed using a X-ray fluroescence ARL PERFORM-X 4200 spectrometer. The compressive tests were carried out on a universal testing machine of 2000 kN.
Data format	Raw and Analyzed
Parameters for data collection	Data presented are formed by thermomechanical damage values, stress versus strains at various temperatures.
Description of data collection	- Nsimalen thermogravimetric and thermal differential analysis (DTA/TGA), - Nsimalen X-Ray Diffraction (XRD) mineralogical analysis, - Nsimalen TM damage values for *b* = 3/2, at different temperatures, - Nsimalen normalized W0 and m for *b* = 5/16 and *b* = 7/16 respectively, - Excel solver was used to carry out numerical calculations.
Data source location	Laboratory of Civil Engineering and Mechanics, National Advanced School of Engineering of Yaoundé, University of Yaoundé 1, P.O. BOX 8390 Yaoundé, Cameroon
Data accessibility	All the data related are within the article.
Related research article	Léon Arnaud Mpoung, Jean Calvin Bidoung, Jean Valdez Sontia Metekong, Jean Raymond Lucien Meva'a, A coupled thermo-mechanical damage model for fired clay bricks based on the unified strength theory, Heliyon 10.1016/j.heliyon.2021e06010

## Value of the Data

• This data can help to improve clay bricks production process through the definition of appropriate firing and operating/use temperatures.
• This data can provide insight for studying the macroscopic response from damage propagation of the clay in the future.
• This data can provide insights to an enhanced understanding of high temperature clay mechanics and provide the basis to improve clay bricks resource durability

## 1 Data Description

**Fig. 1:**
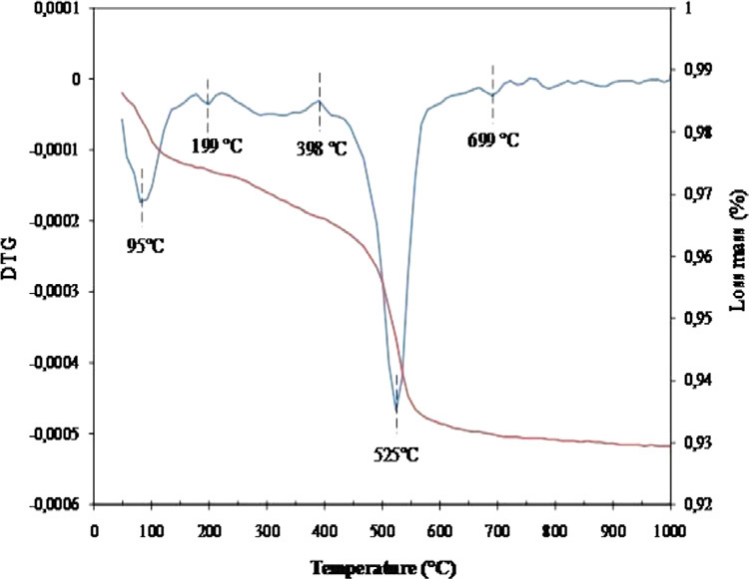
DTA/TGA of clay.

**Fig. 2:**
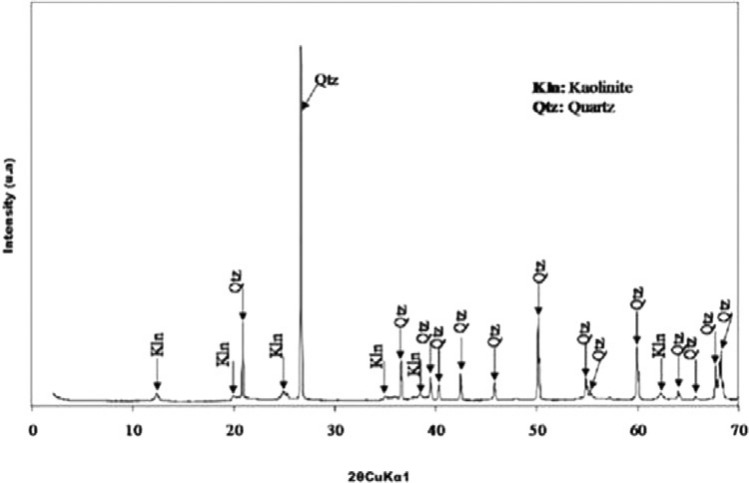
X-ray diffraction peak clay.

**Fig. 3:**
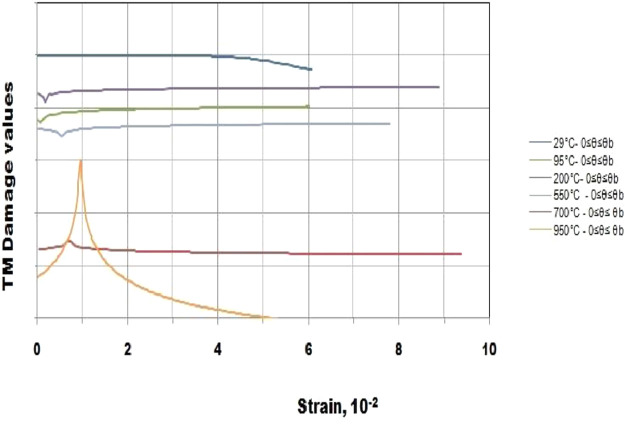
TM Damage values for the effect of the intermediate principal shear stress *b* = 3/2 at different temperatures namely 29, 95, 200, 550, 700 and 950 °C with θ, the stress angle corresponding to the twin-shear parameter of shear stress $0\leq \theta \leq \theta_{b}.$

**Fig. 4:**
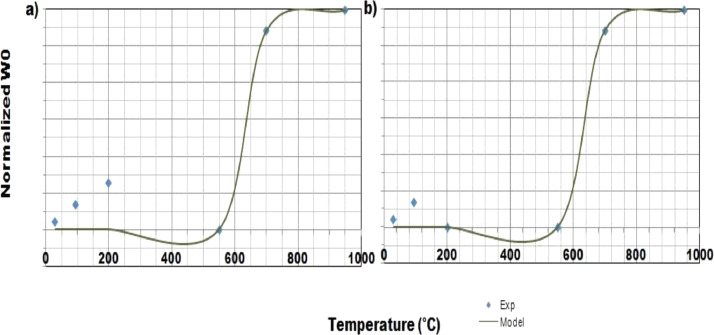
Normalized W0 with temperature for *b* = 5/16 with θ, the stress angle corresponding to the twin-shear parameter of shear stress such that a) $0\leq \theta \leq \theta_{b}$, b)$\;\theta_{b}\leq \theta \leq 60^{\circ }$.

**Fig. 5:**
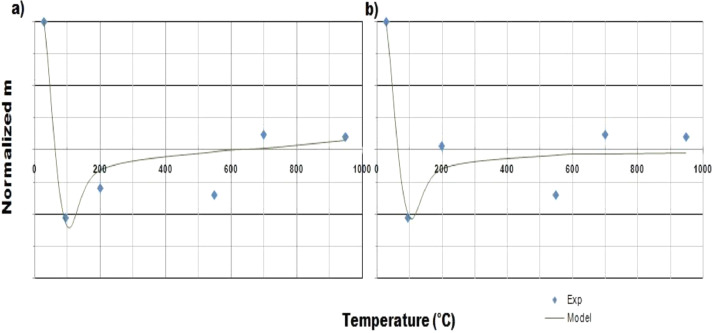
Normalized m with temperature for *b* = 7/16 withθ, the stress angle corresponding to the twin-shear parameter of shear stress such that a)$\;0\leq \theta \leq \theta_{b}$, b) $\theta_{b}\leq \theta \leq 60^{\circ }$.

This article consists of tables and figures that depict data obtained from various tests among other the thermogravimetric and thermal differential analysis (DTA/TGA) (Fig. 1), the X-Ray Diffraction (XRD) (Fig. 2) for thermal and mineralogical analysis respectively of the clay used. Mineralogical analyses have also been made in order to identify the mineral phases (Table 1). Fig. 3 represents the TM Damage values for the effect of the intermediate principal shear stress, *b* = 3/2, of that clay at different temperatures. Meanwhile, Figs. 4 and 5 present the variation of the two distribution/model parameters with respect to the temperature for two values of the effect of the intermediate principal shear stress, *b* = 5/16 and *b* = 7/16 respectively.

**Table 1:** Identified mineral phases.

Identified Phases	d-spacing (Å)
Kaolinite	7.13 ; 4.45 ; 3.57 ; 2.56 ; 2.49 ; 2.34 ; 1.97 ; 1.49
Quartz	3.34 ; 4.25 ; 2.45 ; 2.28 ; 2.23 ; 2.12 ; 1.97 ; 1.81 ; 1.67 ; 1.45 ; 1.41 ; 1.38 ; 1.37

The database is composed of five Excel files that contain raw data (Table 2). It includes data on thermogravimetric and thermal differential analysis (DTA/TGA), X-Ray Diffraction (XRD), thermal and mineralogical analysis, TM Damage values for the effect of the intermediate principal shear stress, fluctuation of the two distribution/model parameters as regards the temperature for two values of the effect of the intermediate principal shear stress.

**Table 2:** Contents of the database.

File name	Variable name	Contents
ATG-ATD_Nsimalen.xls		
	Temperature	ATG-ATD of Nsimalen clay
	Mass	
	Loss mass	
	Calculated derivative	
TM damage value.xls		
	TM damage	Damage of Nsimalen clay
	Strain	
	Temperature	
DRX_Nsimalen.xls		
	Theta (Ѳ)	DRX of Nsimalen clay
	Intensity	
Normalized m		
	Temperature	Progression of normalized m
	Normalized values of m	in relation to temperature
	Experimental value	
Normalized W0		
	Temperature	Trend of normalized W0
	Normalized values of W0	in terms of temperature
	Experimental value	

## 2 Experimental Design, Materials and Methods

Clay from Nsimalen, a locality around Yaoundé (Cameroon), was selected because of its matter of development implementations [2].

The thermogravimetric and thermal differential analysis (TGA/DTA) and the XRD have been undertaken to pinpoint its thermal behavior and mineralogical composition.

Approximately 1 g of material is collected and dried in the oven at 40 °C for at least 24 h. The sample is crushed manually with agate mortar and sieved dry to 250 µm until full passage. An aliquot is placed door by simple pressure in order to limit any preferential orientation of the mineral according to the method of Moore and Reynolds (1997). The resulting difractogram allows the identification of all reflections.

The perculiarity X-ray diffraction (XRD) high points were registered in the progression 2 ° ≤ 2θ ≤ 70 ° with a diffractometer Bruker Advance Eco outfitted with a Cu Kα1 (λ = 1,5418 Å) irradiation generator. The ray generator was exploited at ambiant temperature with tension and current setting as 40 kV and 30 mA respectively.

The thermogravimetric and thermal differential analysis (TG/DTA) was conducted on the Automatic Multiple Sample Thermogravimetric Analyzer TGA-2000 of AGES in an intense airing environment at a warm-up speed of 5 °C/min for 25 ~ 1000 °C ± 1 °C temperature on Nsimalen samples (0.1–1.5 g).

The clay was crushed in a grinder and sieved at 800 µm. Then, the test brick slip were formed as conventional cube-shaped of 4 × 4 × 4 cm operating with combined clay and suitable amount of water (approximately 20% on dry basis) to fulfill right usability. The realized cubes were first desiccated at atmospheric environment for 72 h and positioned in an oven at 105 °C for 24 h for an entire elimination of hydrating water, before being fired in a smoother boiler up to 1050 °C. Five temperatures, to which the samples laid in the furnace are warmed up, were selected: 95, 200, 550, 700 and 950 °C. The compressive tests were accomplished on a universal testing machine of 2000 kN according to ASTM C67–80a standard. The tests were undertaken at a steady rate of motion of 5 mm/min to obtain the absolute stress – strain curves. An equally dispersed and continuously growing charge was required up to specimen breakdown, on cross-sectional sides portrayed by the width and the depth of the brick (4 × 4 × 4 cm).

A coupled thermo-mechanical (TM) damage evolution model [1], [2], [3], [4], [5], [6], [7] was proposed built on a prefered rock Unified Strength Theory (UST) [8], [9] failure criterion. The model approval [1] was implemented through compressive stress-strain data which were fitted by Eq. (1) obtained using a minimization of the square of the error technique.

(1) $$\sigma_{ij}=\mu E_{T}\left(\varepsilon_{ij}-\varepsilon^{th}\right)\left(1-\zeta +\zeta exp\left[-{\left(\frac{W}{W_{0}}\right)}^{m}\right]\right)$$

With

- $\sigma_{ij}$ is the stress component,
- $\varepsilon_{ij}$ is the strain component,
- $\varepsilon^{th}$ is the thermal strain component,
- ζ is the adjustment factor
- μ is the ratio due to the behavior of clay at elevated temperatures
- $E_{T}$ is the modulus of elasticity at temperature T
- W is a Weibull parameter that specifies the constraint level of the submicroscopic constituent,
- $W_{0}\;\left(\text{the}\;\text{scope}\;\text{ratio}\;\text{corresponding}\;\text{to}\;\text{the}\;\text{submicroscopic}\;\text{constraint}\;\text{component}\;\text{part}\right)$ and m (the scope ratio determining the amount of regularity) are the statistical parameter used in the Weibull distribution.

Graphs were produced using Excel after testing functions that could fitted the data obtained.The functions that fitted the data are given by Eqs. (2) and (3) for normalized W and normalized m respectively.

(2) $$\left\{ \begin{array}{c} \frac{W\left(T\right)}{W\left(29{\;}^{\circ }C\right)}=\frac{29.7424}{1+exp\left(-\left(72.171T-42.2266\right)\right)};R^{2}=0.942;\;0\leq \theta \leq \theta_{b}\;\\ \frac{W\left(T\right)}{W\left(29^{\circ }C\right)}=\frac{29.7676}{1+exp\left(-\left(67.2894T-44.81\right)\right)};R^{2}=0.9872;\;\theta_{b}\leq \theta \leq 60^{\circ }\; \end{array}\right.$$

(3) $$\left\{ \begin{array}{c} \frac{m\left(T\right)}{m\left(29{\;}^{\circ }C\right)}=-0.06461\tan\left(-2.68\left(\frac{T}{1000}\right)+1.7125\right);R^{2}=0.9044;\;0\leq \theta \leq \theta_{b}\;\\ \frac{m\left(T\right)}{m\left(29{\;}^{\circ }C\right)}=-0.0012\tan\left(-0.056\left(\frac{T}{1000}\right)+1.573\right);R^{2}=0.883;\;\theta_{b}\leq \theta \leq 60{\;}^{\circ }\; \end{array}\right.$$

## CRediT Author Statement

**Meva'a Jean Raymond Lucien:** Project administration, Conceptualization, Methodology, writing – original draft, validation; **Bidoung Jean Calvin:** Conceptualization, Methodology, Formal analysis, data analysis and interpretation the data, writing – original draft; **Sontia Metekong Jean Valdez:** Resources, Investigation, writing – original draft; **Suila Yilaren**Yilaren**g Basil:** Resources, writing – original draft, writing –reviewing &editing; **Mpoung Léon Arnaud:** Investigation, Formal analysis, Conceptualization, Methodology, analysis tool or data, wrote the paper.

## Declaration of Competing Interest

The authors sincerely thank the Local Materials Promotion Authority (MIPROMALO) in Cameroon for its collaboration in making available the samples used for this study. They equally express their gratitude to the National Civil Engineering Laboratory (LABOGENIE) in Cameroon and the Laboratoire de l'Unité de Recherche Argiles, Géochimie et Environnements sédimentaires (AGEs) de l'Université de Liège where all the tests were carried out.

## Appendix Supplementary materials

Image, application 1
